# Supplementary material for: Identification of photoperiod-induced specific miRNAs in the adrenal glands of Sunite sheep (Ovis aries)
Source: Front Vet Sci. 2022 Jul 22;9:888207. doi: 10.3389/fvets.2022.888207 (PMC9354845; doi:10.3389/fvets.2022.888207)
Supplement: Supplementary file 1 [file Data_Sheet_1.ZIP › Supplementary file/miRDeep2 file.zip!/miRDeep2 file/Novel_Structure/SP42A3/Novel_518.pdf]

| | | |
|---|---|---|
| Provisional ID | : | NC_019484.2_32756 |
| Score total | : | 2 |
| Score for star read(s) | : | -1.3 |
| Score for read counts | : | 0 |
| Score for mfe | : | 1.7 |
| Score for randfold | : | 1.6 |
| Score for cons. seed | : | |
| Total read count | : | 26 |
| Mature read count | : | 26 |
| Loop read count | : | 0 |
| Star read count | : | 0 |

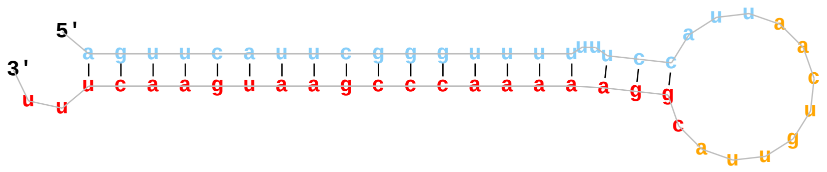


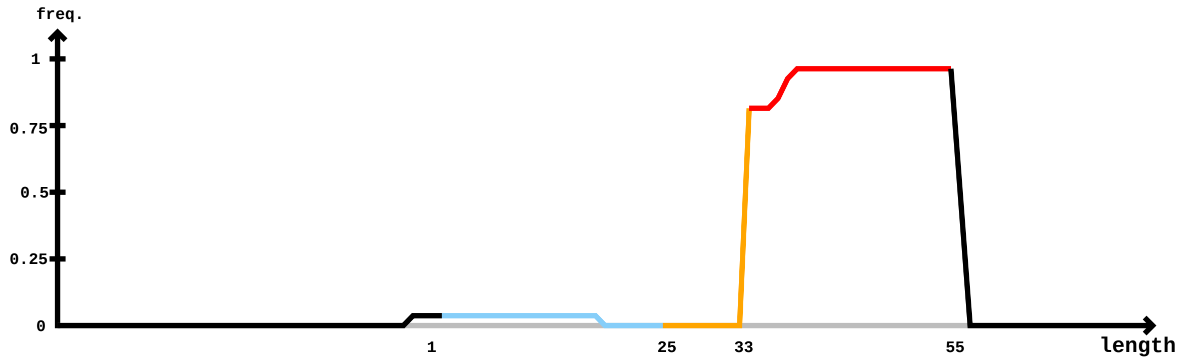


Star Mature

```
5'- cuuccaagaaagagacaauguuccaugguuggccaaaaaguucauucggguuuuuuuccauuaacuguuacggaaaaacccgaaugaacuuucugcccaacccaguauuauc   -3'    exp
    .........................((((((.((.((((((((((((((((((..(((............))))))))))))))))))))).)).))))))..........    reads      mm         sample
    ..................................aaaaguucauucggguuuuu..........................................................    1          0          seq
    ......................................................................Uggaaaaacccgaaugaacuuu....................    21         1          seq
    ......................................................................cggaaaaacccgaaugaacuuu....................    1          0          seq
    .........................................................................aaaaacccAaaugaacuuu....................    1          1          seq
    ..........................................................................aaaacccAaaugaacuuu....................    1          1          seq
    ..........................................................................aaaaccUgaaugaacuuuc...................    1          1          seq
    ...........................................................................aaacccgaaugaacuuu....................    1          0          seq
```
